# Kcnh2 mediates FAK/AKT‐FOXO3A pathway to attenuate sepsis‐induced cardiac dysfunction

**DOI:** 10.1111/cpr.12962

**Published:** 2020-12-02

**Authors:** Zhigang Li, Yilei Meng, Chang Liu, Huan Liu, Wenze Cao, Chang Tong, Min Lu, Li Li, Luying Peng

**Affiliations:** ^1^ Key Laboratory of Arrhythmias Ministry of Education Shanghai East Hospital Tongji University School of Medicine Shanghai China; ^2^ Institute of Medical Genetics Tongji University Shanghai China; ^3^ Heart Health Center Shanghai East Hospital Tongji University School of Medicine Shanghai China; ^4^ Department of Medical Genetics Tongji University School of Medicine Shanghai China; ^5^ Research Units of Origin and Regulation of Heart Rhythm Chinese Academy of Medical Sciences Beijing China

**Keywords:** AKT, apoptosis, FOXO3A, Kcnh2, sepsis‐induced cardiac dysfunction

## Abstract

**Objectives:**

Myocardial dysfunction is a significant manifestation in sepsis, which results in high mortality. Even Kcnh2 has been hinted to associate with the pathological process, its involved signalling is still elusive.

**Materials and methods:**

The caecal ligation puncture (CLP) surgery or lipopolysaccharide (LPS) injection was performed to induce septic cardiac dysfunction. Western blotting was used to determine KCNH2 expression. Cardiac function was examined by echocardiography 6 hours after CLP and LPS injection in Kcnh2 knockout (Kcnh2^+/‐^) and NS1643 injection rats (n ≥ 6/group). Survival was monitored following CLP‐induced sepsis (n ≥ 8/group).

**Results:**

Sepsis could downregulate KCNH2 level in the rat heart, as well as in LPS‐stimulated cardiomyocytes but not cardiac fibroblast. Defect of Kcnh2 (Kcnh2^+/‐^) significantly aggravated septic cardiac dysfunction, exacerbated tissue damage and increased apoptosis under LPS challenge. Fractional shortening and ejection fraction values were significantly decreased in Kcnh2^+/‐^ group than Kcnh2^+/+^ group. Survival outcome in Kcnh2^+/‐^ septic rats was markedly deteriorated, compared with Kcnh2^+/+^ rats. Activated Kcnh2 with NS1643, however, resulted in opposite effects. Lack of Kcnh2 caused inhibition of FAK/AKT signalling, reflecting in an upregulation for FOXO3A and its downstream targets, which eventually induced cardiomyocyte apoptosis and heart tissue damage. Either activation of AKT by activator or knockdown of FOXO3A with si‐RNA remarkably attenuated the pathological manifestations that Kcnh2 defect mediated.

**Conclusion:**

Kcnh2 plays a protection role in sepsis‐induced cardiac dysfunction (SCID) via regulating FAK/AKT‐FOXO3A to block LPS‐induced myocardium apoptosis, indicating a potential effect of the potassium channels in pathophysiology of SCID.

## Graphical abstract

Schematic representation shows the mechanisms of Kcnh2‐modulated cardiac dysfunction following sepsis stimulus. Sepsis‐induced decrease of Kcnh2 resulted in inhibition of AKT in cardiomyocytes. Attenuation of AKT of cardiomyocytes mediated by Kcnh2 upregulated FOXO3A expression, which initiated the transcription of BIM/PUMA genes. Finally, enhanced BIM/PUMA caused the cardiomyocytes apoptosis, leading to cardiac dysfunction.

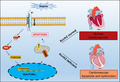

## 1 INTRODUCTION

Highly concerning is always drawn on sepsis, a major cause of death in the intensive care unit, which can attribute to uncontrolled inflammatory responses, mitochondrial energy metabolism disorders and cell apoptosis.^1^, ^2^, ^3^ Particularly, sepsis‐induced cardiac dysfunction (SICD) is associated with significant mortality, which clinically manifests myocardium damage due to cardiomyocyte apoptosis.^4^, ^5^, ^6^, ^7^ However, its pathogenesis is still not fully understood.

The potassium voltage‐gated channel subfamily H member (KCNH), 6‐transmembrane–spanning and one‐pore–forming domain protein, is generally responsible for the excitability of tissues.^8^ Kcnh2 (hERG1 or Kv11.1) is highly expressed in heart, and its mutation is often associated with arrhythmias.^9^ Recently, KCNH2 channels are also found to involve in modulating cell proliferation and apoptosis.^10^ For example, mutant KCNH2 with homozygous missense N629D or inhibition of the channel by small molecule doxazosin really can induce cellular apoptosis both in vivo and vitro.^11^, ^12^ KCNH2 channels have also been identified as a significant effector in sepsis‐induced atrial tachyarrhythmias.^13^, ^14^ In addition, IL‐6 impaired KCNH2 channel currents in cardiomyocytes by downregulating KCNH2 alone or in combination with the soluble IL‐6 receptor (IL‐6R).^15^ However, no evidence is available how Kcnh2 modulates sepsis‐related apoptosis in heart.

Phosphoinositide 3‐kinase(PI3K)/ serine/threonine kinase AKT (protein kinase B) regulates a wide range of physiological processes, including inflammatory responses and apoptosis.^16^ Activation of PI3K/AKT can prevent against LPS‐induced acute inflammatory injury through limiting pro‐inflammatory and apoptotic events.^17^, ^18^ In turn, inhibition of PI3K/AKT deteriorates the inflammatory damage via upregulating pro‐inflammatory Transcription factors (TFs), such as NF‐κB, which next promotes the release of inflammation cytokines.^18^, ^19^ It has been known that Kcnh2 could modulate AKT signalling by interacting with integrin β1.^20^ Activation of AKT inhibits FOXO3A expression, resulting in reducing apoptosis by decreasing transcriptionally activating the pro‐apoptosis genes BIM/PUMA.^16^, ^21^ Moreover, cardiac‐specific overexpression of FOXO3A induces a decrease of heart weight by reducing individual cardiomyocyte size.^22^ FOXO3A also drives the expression of BNIP3 based on regulation of JNK signalling, which further induces mitochondrial apoptosis and mitophagy in heart failure.^23^ These evidences strongly suggest that Kcnh2 may mediate AKT/FOXO3A pathways to modulate the sepsis‐induced pathological process in heart.

Here, the investigation was performed using rat models of sepsis induced by CLP surgery or LPS injection. Transgenic rats with heterozygous deficiency and NS1643‐treated were used to decrease or active KCNH2. These studies suggest that Kcnh2 plays a protective role in SCID by reducing cardiomyocyte apoptosis through AKT/FOXO3A/BIM pathways, providing rationale for Kcnh2 as a promising candidate target in SCID.

## 2 METHODS

### 2.1 Animal

To generate the rat Kcnh2 knockout model, the CAG‐GFP‐IRES‐Pac was inserted to replace the exon 7‐8 by homologous recombination. Targeting construct and genotyping method were done as supplementary data (Figure S1). Male Sprague Dawley rats weight between 220 g and 250 g were purchased from Slaccas Company and kept in the animal facility at Tongji University. All of the procedures were approved by Institutional Animal Care and Use Committee at Tongji University (approval no: TJLAC‐016‐022). All of animal experiments were performed in accordance with the National Institutes of Health Guide for the Care and Use of Laboratory Animals (NIH Publication No. 85‐23, revised 1996).

### 2.2 Sepsis model

#### 2.2.1 Caecal ligation and puncture surgery model

Male rats weight between 220 g and 250 g were administered CLP surgery as previous reported with part modification.^24^ Rats were anaesthetized with intraperitoneal injection of mixture of ketamine (100 mg/kg) and xylazine (25 mg/kg). Make a 2‐3 cm incision at the midline of the abdomen to expose the caecum, which was ligated with a 4‐0 silk 1 cm from the end of the caecum distal to the intestine and punctured through‐and‐through with an 18‐gauge needle. A small amount of faecal contents was gently squeezed through the puncture site. The bowel was then situated back in the abdomen and the incision was sutured with a sterile 4‐0 silk suture. The sham group underwent the same procedure except caecal ligation and puncture. All animals were kept in 37℃ and water immediately after the surgery.

#### 2.2.2 LPS‐induced model

For LPS endotoxaemia, rats were administered i.p. with either lethal (10 mg/kg) or sublethal (4 mg/kg) doses of LPS derived from E. coli 0111: B4 (Sigma‐Aldrich) as previously reported.^25^ Sterile PBS was used as vehicle control in sham groups.

At the time of sacrifice, all rats were euthanized by inhaling in CO_2_, the euthanasia method used for all animal procedures.

### 2.3 Echocardiography

Six hours after CLP surgery or LPS injection, rats were performed transthoracic echocardiograms using Vevo770 small animal echocardiography machine (Visual Sonics Inc.) as previously described.^26^ Three consecutive cardiac cycles each checking were calculated for cardiac function analysis. LV ejection fraction (EF %) and LV fractional shortening (FS %) were calculated. All of rats were performed under anaesthesia (1.5%‐2% isoflurane, 2 L/min oxygen flow rate).

### 2.4 Histopathology

Fix the fresh tissues with 10% paraformaldehyde and embed it in paraffin by standard protocols. Then, the samples were sectioned at 8 μm; haematoxylin and eosin (HE) and immunohistochemical staining were performed. A rabbit monoclonal antibody against p‐AKT (Abcam, catalogue number ab81283; 1:250) and a rabbit monoclonal antibody against p‐FAK (Abcam, catalogue number ab81298; 1:250) were incubated overnight. The slides were then observed under a Leica confocal microscope.

### 2.5 Cell culture and treatment

Neonatal Sprague Dawley rats born within 24 hours were purchased from Slaccas Company. Isolation and culture of primary neonatal rat cardiomyocytes (NRCMs) or cardiac fibroblasts (CFs) were performed as previous described.^26^ Cells were cultured in DMEM with 10% foetal bovine serum (FBS), while cardiomyocytes harboured 0.1 mM BrdU (Sigma) for 48 hours. The serum‐free DMEM was used to pre‐treat NRCMs for overnight before indicated experiments.

### 2.6 Western blot

Hearts, NRCMs and CFs were lysed using RIPA to extract the total protein and concentration of protein was determined using a BCA Protein Assay kit (TaKaRa). The samples were analysed with 8%‐15% SDS‐PAGE gels and transferred to 0.45‐μm PVDF membranes (Millipore). The membranes were blocked with 5% BSA and incubated with primary antibodies: KCNH2 (ab136467; 1:1000), FOXO3A (ab17026; 1:1000), BCL‐2 (ab59348; 1:1000), p‐AKT (ab81283; 1:1000), AKT (ab179463; 1:1000), p‐FAK(ab81298; 1:1000) and FAK (ab40794; 1:1000) (Abcam); BIM (Cell Signaling Technology, 2933T, 1:1000); PUMA (Santa Cruz Biotechnology, USA,sc‐374223, 1:100); GAPDH (Proteintech, 60004‐1‐lg, 1:1000) was applied as a loading control. The membranes were then incubated with secondary antibodies anti‐mouse (sa00001‐1), anti‐rabbit (sa00001‐2) (Proteintech) and imaged with Amersham Imager 600 or ImageQuant LAS 4000.

### 2.7 RNA extraction and quantification

Total RNA was extracted from NRCMs and rat heart tissues using TRIzol reagent (Invitrogen) and was converted to cDNA by applying PrimeScript™ RT reagent Kit with gDNA Eraser (TaKaRa). Then, expression of genes was determined by applying TB Green™ Premix Ex Taq™ (TaKaRa) on a Bio‐Rad CFX connect™ real‐time PCR system using the primers:

Kcnh2 forward primer: 5′‐GATCGGCAAGCCCTACAACA‐3′ and Kcnh2 reverse primer: 5′‐ GAGCGCTGTGACGTACTTGT‐3′; GAPDH forward primer: 5′‐AAGGTCGGTGTGAACGGATT‐3′ and GAPDH reverse primer: 5′‐CTTTGTCACAAGAGAAGGCAGC‐3′.

### 2.8 Si‐RNA transfection

Neonatal rat cardiomyocytes were seeded at 2 × 10^6^ in 6‐well plates or at 4 × 10^4^ in 24‐well plates. After starving serum‐free DMEM cultured overnight, si‐RNA was transfected using lipofectamine 2000 (Invitrogen) at a terminal concentration of 20 nM. 4‐6 hours later, NRCMs were changed with fresh medium and cultured for another 24‐48 hours before conducting next experiments. Si‐FOXO3A: 5′‐CUCUAUAACGUAUGCAAAU‐3′ and Scrambled si‐RNA were purchased by Genepharma.

### 2.9 TUNEL assay

Apoptosis rate of heart sections and NRCMs was determined by terminal deoxynucleotidyl transferase(TdT)‐mediated dUTP nick end‐labelling (TUNEL) assay (Roche). The samples were fixed and permeated, followed with adding 80 μL of TUNEL reaction mixture onto samples for 60 minutes at 37°C in dark, and then, the samples were incubated with Hoechst for 12 minutes at RT and finally imaged using Leica confocal microscope system.

### 2.10 ELISA

Rat blood serum was collected from rats by retro‐orbital bleeding and plasma was isolated using lithium heparin‐coated plasma separator tubes (BD) according to the manufacturer's instructions. Inflammatory cytokines in the serum were measured 6 hours after surgery by using commercially available IL‐1β (Proteintech KE20005) and TNF‐α (SAB, EK0517) ELISA kits and the concentration were analysed on a Bio‐Rad microplate reader system.

### 2.11 Statistical analysis

ANOVA test was used to compare among three or more groups, followed by Turkey's post hoc test. Student's *t* test was applied to compare two groups, and the error bar represented the standard error of mean (SEM). A value of *P* < .05 was considered significant. All data were analysed using Prism 5.0 (GraphPad Software, Inc).

## 3 RESULTS

### 3.1 Kcnh2 was downregulated in septic heart and cardiomyocytes

**Figure 1:**
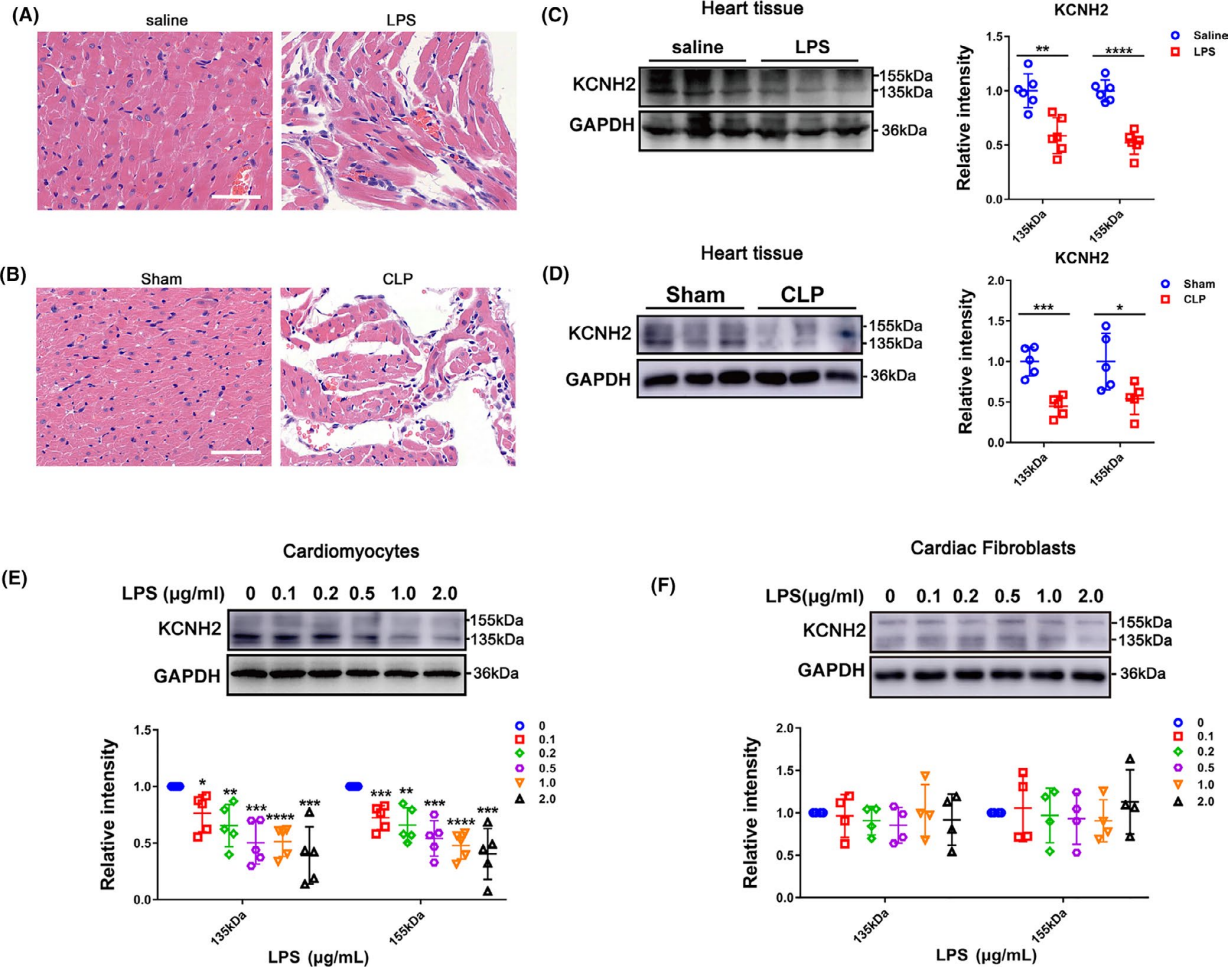
Sepsis reduces myocardial Kcnh2 expression. For LPS‐induced rat model (A) and CLP rat model (B), haematoxylin–eosin staining (H&E staining) was performed to detect heart injury. Scale bar: 50 μm. After stimulation for 6 h, KCNH2 expression was immunoblotted in rat heart of (C) LPS model (n = 6) and (D) CLP model (n = 5). (E) The expression of KCNH2 was showed in cultured cardiomyocytes and (F) cardiac fibroblasts by Western blot at 12 h after either saline or LPS treatment at different concentration (0, 0.1, 0.2, 0.5, 1.0, 2.0 μg/mL), (n ≥ 4). (These results were performed as mean ± SD of at least 4 independent experiments. Statistical analysis was performed with one‐way ANOVA followed by Tukey's test. *, *P* < .05; **, *P* < .01; ***, *P* < .001 and ****, *P* < .0001)

To explore the effect of Kcnh2 on septic heart and cardiomyocytes, we detected the expression level of Kcnh2 in SICD, we applied CLP surgery to prepare a rat model with polymicrobial sepsis or induced endotoxaemia by intraperitoneal injection of bacterial LPS, which resulted in a typical feature of sepsis‐lung injury (Figure S2). Sepsis led to significant cardiac damages such as diffuse interstitial oedema, hyperaemia, haemorrhages, cardiomyocyte degeneration with myofibril lysis, lost cross striations in most myofibril and obvious cellular infiltration (Figure 1A,B). The mRNA and protein level of Kcnh2 were found to be significantly downregulated in septic heart tissues, compared with control rats (Figure S3A,B, Figure 1C,D). Furthermore, we challenged primary neonatal rat cardiomyocytes (NRCMs) and cardiac fibroblasts (CFs) with LPS at different concentrations. Kcnh2 expression was found to be inhibited with a concentration‐dependent pattern in cardiomyocytes in presence of LPS, which was consistent with that observed in heart tissues (Figure S3C, Figure 1E). However, there is no significant difference in CFs after LPS stimulation (Figure 1F). The in vivo and in vitro results indicated that Kcnh2 was really regulated in cardiomyocytes during SICD process.

### 3.2 Loss of Kcnh2 aggravates the heart damage in SICD models

**Figure 2:**
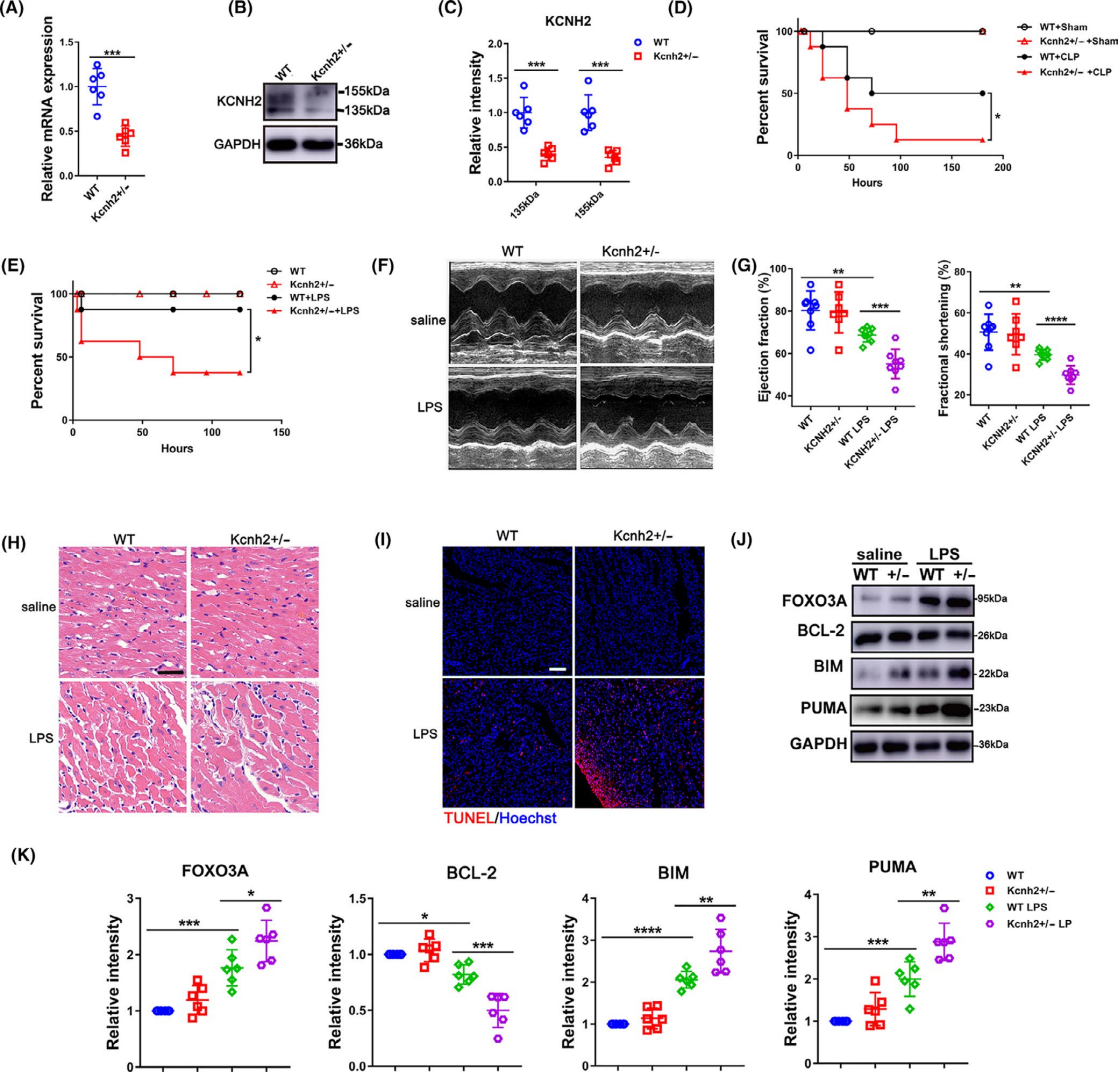
Kcnh2 defect promotes the heart damage induced by sepsis. The levels of (A) mRNA of Kcnh2 and (B‐C) protein of KCNH2 in WT or Kcnh2^+/‐^ cardiac extracts, (n = 6). (D) Survival status of Kcnh2^+/‐^ and WT rats after CLP surgery (n = 8) or (E) after LPS stimulation (n = 8). The change of heart function in Kcnh2^+/‐^ and WT rats at 6 h after LPS challenge, (F‐G) left ventricular ejection fraction (FS %) and fractional shortening (EF %) measured with echocardiography, (n = 8); (H) H&E staining for detecting the heart tissue damage, (n = 5). (I) The cardiac apoptosis determined by TUNEL assay (n = 5), scale bar: 75 μm. (J‐K) Western blot and quantitative analysis for FOXO3A, BCL‐2, BIM and PUMA in cardiac extracts (n = 6). (All the results were performed as mean ± SD of at least 6 independent experiments. Statistical analysis was performed with one‐way ANOVA followed by Tukey's test. *, *P* < .05; **, *P* < .01; ***, *P* < .001 and ****, *P* < .0001)

Reduced expression of Kcnh2 suggested its potential role in SICD. To further explore the effects of Kcnh2, we confirmed the status of Kcnh2 expression and performed electrocardiogram (ECG) for Kcnh2^+/‐^ rats. We found the expression of Kcnh2 was significantly decreased and the defect of KCNH2 also led to significant prolong of QTs interval (Figure 2A‐C, Figure S4). Then, the CLP surgery was used to induce sepsis on the Kcnh2^+/‐^ rats. Our results show that CLP stimulation really made wild‐type (WT) rats have poor survival rate, compared with the sham group and unchallenged Kcnh2^+/‐^ rats. The CLP surgery, however, induced the rats with Kcnh2^+/‐^ genetic background to exhibit lower rate of survival (Figure 2D). A similar survival rate was also observed in LPS‐challenged model (Figure 2E).

Since sepsis could cause multiple organs dysfunction, we next analysed the damage of kidney, liver, lung and spleen stimulated by LPS among WT, Kcnh2^+/‐^ or NS1643, compared with control. As shown in the results, LPS could significantly damage these organs but the heterozygous deficiency or activation of Kcnh2 has no effect on the tissues injury (Figure S5). However, the injury of heart was significantly influenced by Kcnh2. In presence of LPS, the deficiency of Kcnh2 also remarkably aggravated the myocardial tissue damage with an increase of apoptosis (Figure 2H‐I). LPS also induced more serious hypertrophy in Kcnh2^+/‐^ rats compared with that in WT group (Figure S6). However, the fibrosis induced by LPS, which was often a consequence of inflammation, had no any difference (Figure S7).

To further confirm the potential role of Kcnh2 on SICD, we performed serial in vivo echocardiographic (M‐mode) and found that even LPS could lead to a significant reduction in the left ventricular ejection fraction (EF %) and fractional shortening (FS %) for WT rats, but more serious impact on Kcnh2^+/‐^ models, with an almost 20% decrease in EF and 10% reduce in FS, respectively, compared to WT group (Figure 2F, G). Moreover, Kcnh2^+/‐^ rats exhibited a lower expression of anti‐apoptosis gene BCL‐2, and higher expression of pro‐apoptosis genes BIM/PUMA than WT rats induced by LPS (Figure 2J, K). Collectively, Kcnh2 might be a potential target to protect heart from inflammatory injury through regulating FOXO3A.

Mortality in the LPS models is usually considered to be outcome of tissue injury caused by uncontrollable inflammation. Therefore, the result of Kcnh2 deletion might be caused by either enhanced inflammation or attenuated ability of tissues to tolerate inflammatory injury. To evaluate this, we detected plasma levels of TNF‐α and interleukin (IL)‐1β. As the results shown, there was no difference between Kcnh2 deletion and WT rats (Figure S8). These data indicated that the increase of mortality observed with Kcnh2 deletion may be an outcome of reduced ability of tissues to tolerate inflammatory injury, rather than the consequence of an expanded inflammatory response.

### 3.3 Enhancing Kcnh2 activity attenuates the sepsis‐induced heart damage

**Figure 3:**
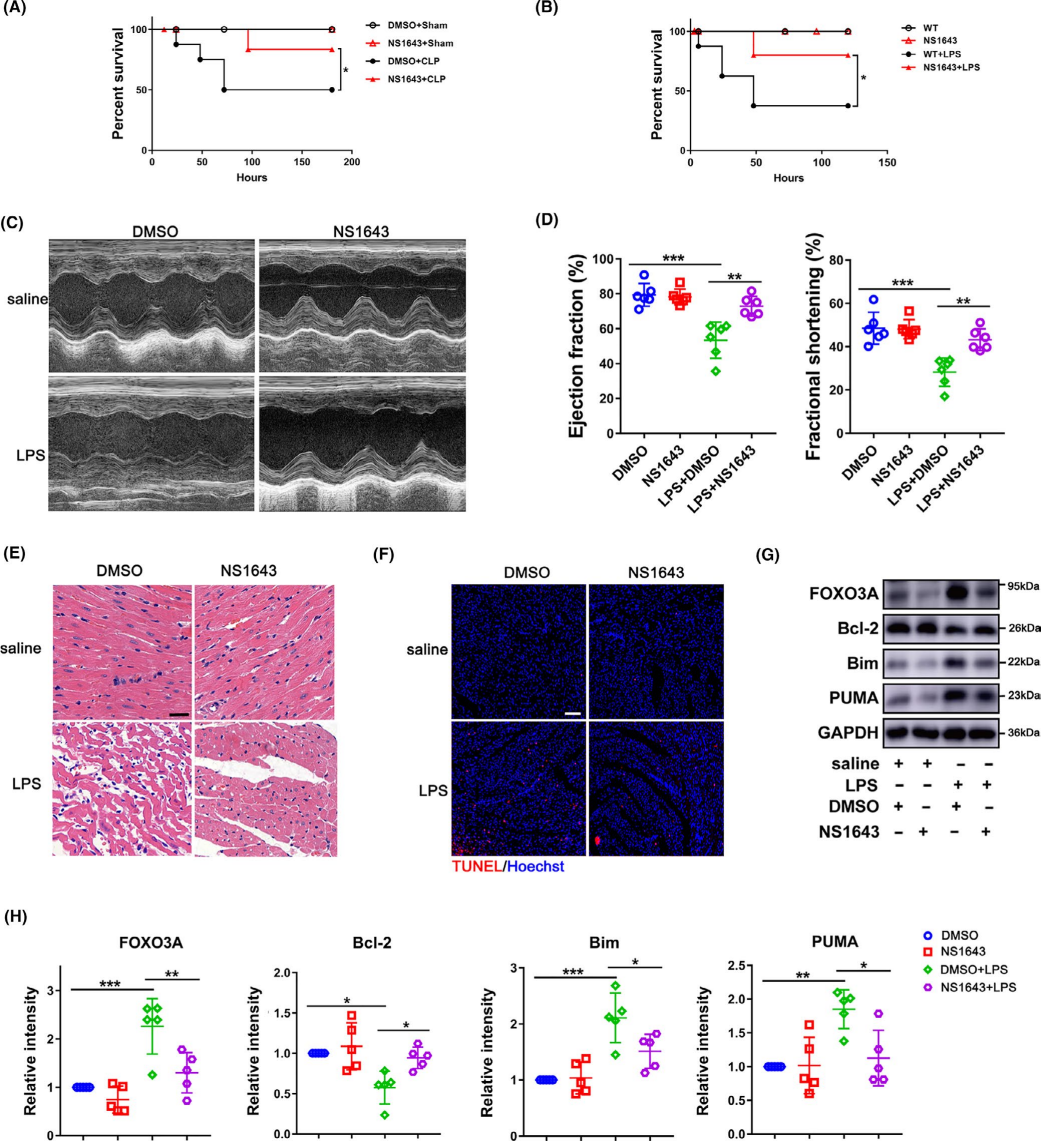
Kcnh2 activator NS1643 attenuates the heart injury induced by sepsis. (A) Survival status after CLP surgery following pre‐treatment of either NS1643 (6 mg/kg) or DMSO only, (n = 8). (B) Survival status after treatment of LPS following pre‐treatment of either NS1643 (6 mg/kg) or DMSO only, (n = 8). (C‐D) The effects of NS1643 on left ventricular ejection fraction (FS %) and fractional shortening (EF %) in presence of LPS, (n = 6); (E) H&E staining of heart sections for detecting the damage of tissue, (n = 5), scale bar: 50 μm. (F) The cardiac apoptosis by TUNEL assay, (n = 5), scale bar: 75 μm. (G‐H) Western blot and quantitative analysis for FOXO3A, BCL‐2, BIM and PUMA in cardiac extracts (n = 5). (All of the results were performed as mean ± SD of at least 5 independent experiments. Statistical analysis was performed with one‐way ANOVA followed by Tukey's test. *, *P* < .05; **, *P* < .01; ***, *P* < .001 and ****, *P* < .0001)

NS1643, a Kcnh2 activator, was applied to activate Kcnh2 for confirming again the role of Kcnh2 in SICD. The Kcnh2 activation with NS1643 really reduced the mortality induced by CLP surgery or LPS challenge (Figure 3A‐B), which could improve the cardiac function, manifesting as increase of the EF % and FS% (Figure 3C‐D), and attenuating the cardiac damage partly due to reduced infiltration of immune cells (Figure 3E). NS1643 also reduced LPS‐induced rat heart apoptosis (Figure 3F), evidenced with an increase of BIM/PUMA expression and a decrease of BCL‐2, accompanied with decreased expression of FOXO3A (Figure 3G‐H). Similar to previous results, the plasma levels of TNF‐α and IL‐1β had no significant difference (Figure S8). These data, hereby, indicate Kcnh2 is crucial to attenuate the SICD via modulating cardiomyocyte apoptosis.

### 3.4 Defect of kcnh2 aggravates the sepsis‐induced cardiomyocyte damage

**Figure 4:**
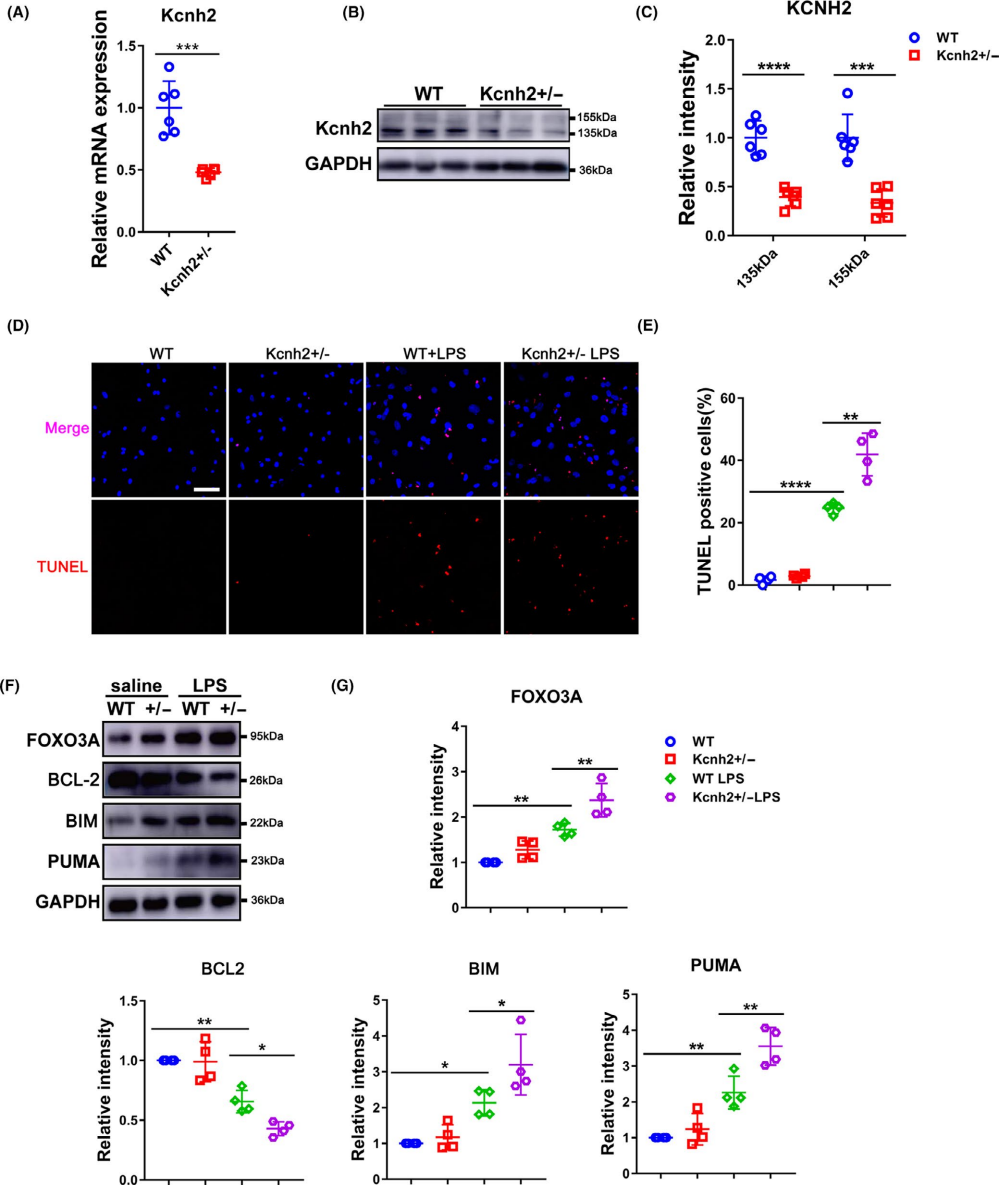
Kcnh2 defect aggravates the LPS‐induced damage in cardiomyocytes. (A ‐C) The Kcnh2 levels of mRNA and protein in WT or Kcnh2^+/‐^ NRCMs extracts, (n = 6). (D‐E) TUNEL assay results for Kcnh2^+/‐^ or WT NRCMs treated with LPS for 24 h, (n = 4). Scale bar: 50 μm. (F‐G) Expression levels for FOXO3A, BCL‐2, BIM and PUMA in Kcnh2^+/‐^ or WT NRCMs in presence of LPS for 24 h, (n = 4). (These results were performed as mean ± SD of at least 4 independent experiments. Statistical analysis was performed with one‐way ANOVA followed by Tukey's test. *, *P* < .05; **, *P* < .01; ***, *P* < .001 and ****, *P* < .0001)

Since the alteration of Kcnh2 expression on a damaged heart was mainly observed in cardiomyocytes, and endotoxin could increase mortality by damaging cardiomyocytes in SICD,^5^ we assessed whether Kcnh2 modulates the fate of cardiomyocytes during sepsis. NRCMs of Kcnh2^+/‐^ and WT rats were isolated and treated with LPS, respectively. Like in vivo results, the low level of Kcnh2 due to its defect in NRCMs (Figure 4A‐C) could also aggravate LPS‐induced cellular apoptosis (Figure 4D‐E), and upregulate FOXO3A that then promoted BIM/PUMA expression. Meanwhile, Kcnh2^+/‐^ made the cardiomyocytes downregulate BCL‐2, which also agreed with the observation in LPS rat models (Figure 4F‐G). It is meaningful that Kcnh2 involves improvement of the cardiac dysfunction in sepsis through modulating cardiomyocyte apoptosis process.

### 3.5 FOXO3A knockdown diminishes the effect of Kcnh2 on LPS‐induced damage

**Figure 5:**
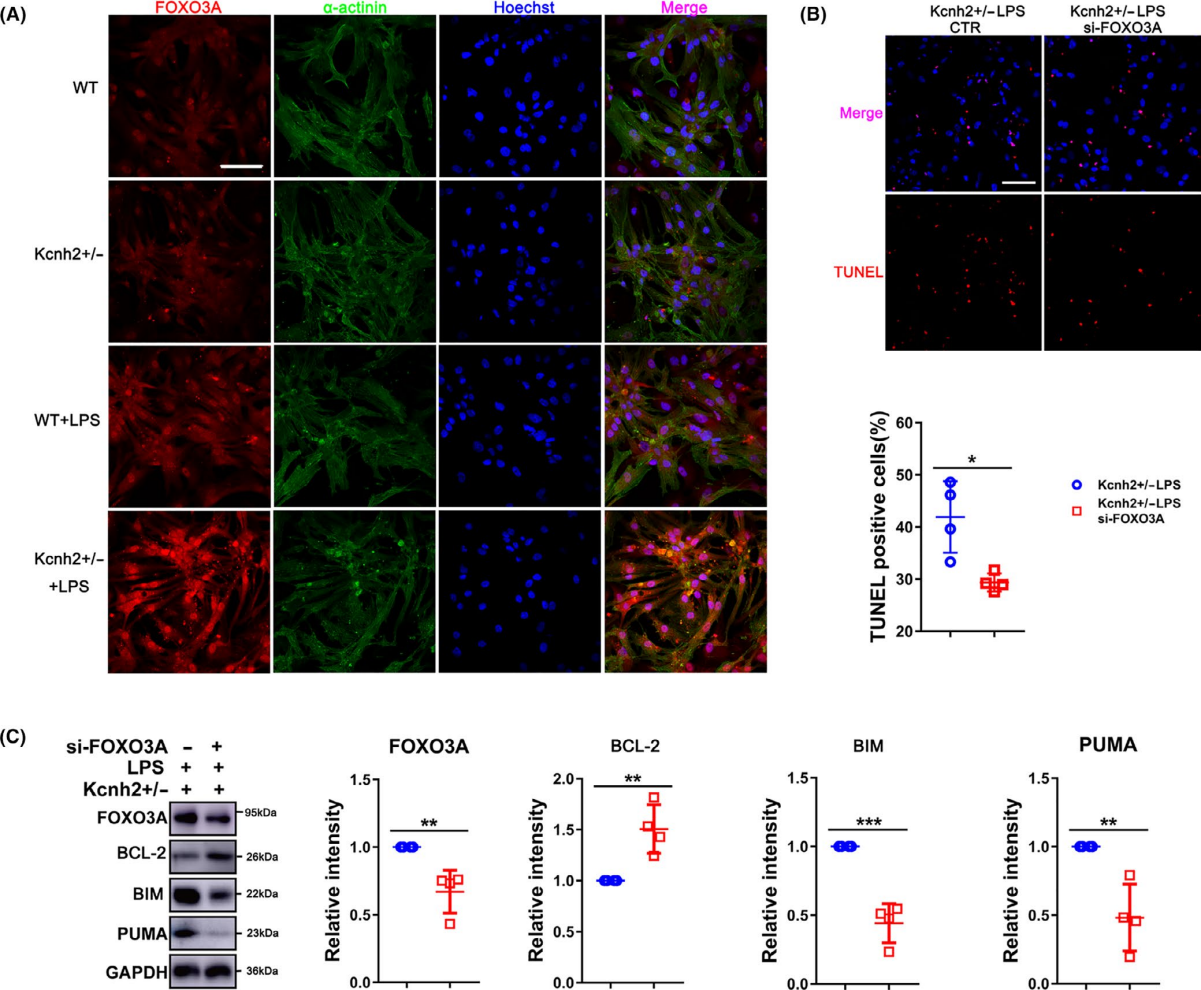
FOXO3A mediates the modulation of Kcnh2 on sepsis‐induced apoptosis. (A) The immunofluorescence was performed to detect the express and location of FOXO3A in Kcnh2^+/‐^ and WT rats at 6 h after LPS challenge. (n = 4). Scale bar: 50 μm. (B) The apoptosis image of Kcnh2^+/‐^ or WT NRCMs after knockdown of FOXO3A by si‐RNA in presence of LPS, (n = 4). Scale bar: 50 μm. (C) Western blot and quantitative analysis for the expression of FOXO3A, BCL‐2, BIM and PUMA in NRCMs after inhibition of FOXO3A under LPS treatment, (n = 4). (All the results were performed as mean ± SD of at least 4 independent experiments. Statistical analysis was performed with one‐way ANOVA followed by Tukey's test. *, *P* < .05; **, *P* < .01; ***, *P* < .001 and ****, *P* < .0001)

Given that SICD could activate FOXO3A/BIM signalling, which has been shown to associate with multiple cellular activities such as stress resistance, apoptosis and cell cycle arrest,^23^ we next ask whether FOXO3A mediates the modulation of Kcnh2 in the pathological process. Immunofluorescence results showed that FOXO3A expression and nuclear retention were significantly increased in LPS‐treated Kcnh2 ^±^ rats, while NS1643 could decrease both expression and nuclear retention of FOXO3A (Figure 5A, Figure S9A‐B). Knockdown assay with specific si‐RNA revealed that inhibition of FOXO3A could significantly reduce the apoptosis of cardiomyocytes, which aggravated due to Kcnh2 defection under LPS challenged (Figure 5B). Western blot analysis further confirmed a remarkable suppression of BIM/PUMA along with the condition, while BCL‐2 level was expectedly enhanced (Figure 5C). Taken together, our data suggest that Kcnh2‐FOXO3A axis really involved in the regulation of cardiomyocyte apoptosis in SICD.

### 3.6 FAK/AKT signalling axis involves in the regulation of Kcnh2 during SICD

**Figure 6:**
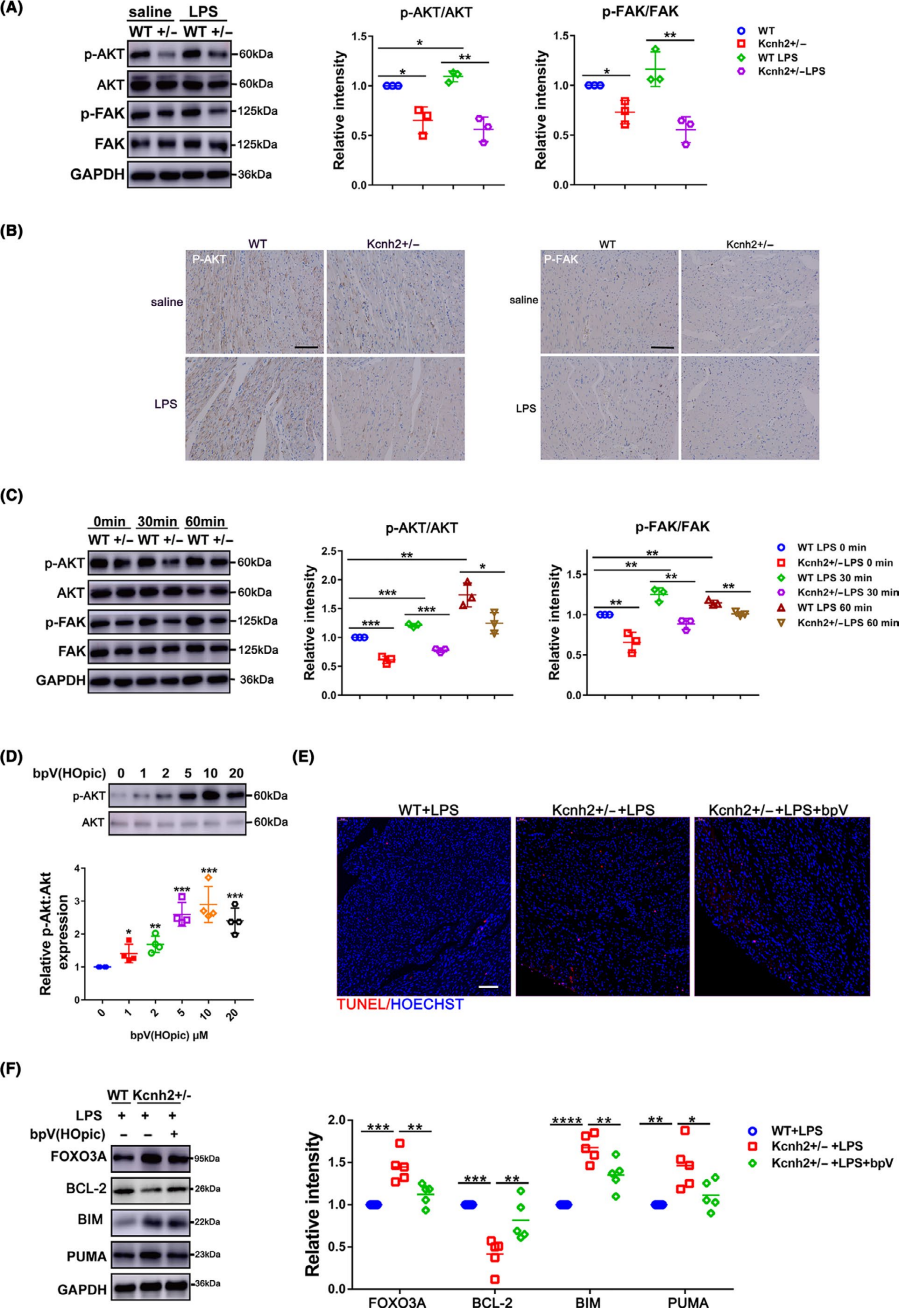
FAK/AKT involves in the regulation of Kcnh2 in sepsis‐induced cardiac apoptosis. (A) The phosphorylation levels of AKT and FAK in Kcnh2^+/‐^ and WT rat cardiac extracts challenged with LPS (n = 3). (B) Immunohistostaining for phosphorylation status of AKT and FAK, (n = 3). Scale bar: 100 μm. (C) The phosphorylation levels of AKT and FAK in cardiomyocytes challenged by LPS at different time points (D) The phosphorylation change of AKT in NRCMs treated with bpV(HOpic).(n = 4). (E) TUNEL staining for heart treated with bpV(HOpic) and LPS, (n = 4), scale bar: 75 μm. (F) Western blot and quantitative analysis for the expression of FOXO3A, BCL‐2, BIM and PUMA in heart treated with bpV(HOpic) and LPS, (n = 5). (These results were performed as mean ± SD of at least 3 independent experiments. Statistical analysis was performed with one‐way ANOVA followed by Tukey's test. *, *P* < .05; **, *P* < .01; ***, *P* < .001 and ****, *P* < .0001)

FOXO3A, as one of the major downstream effectors of AKT, has been shown to be upregulated with LPS challenge in cardiomyocytes.^26^ Here, we found that the deficiency of Kcnh2 simultaneously led to a decrease of AKT activity independent of intergrin β1, which subsequently enhanced the activity of FOXO3A/BIM‐PUMA pathway (Figure 6A, Figure S11). It was worth noting that the phosphorylation of FAK, a direct activator of AKT, shown a change characteristic consistent with AKT, suggesting the signalling axis was really triggered in the disorder condition (Figure 6A).

The immunohistochemical results of in vivo model were further confirmed in the involved cardiomyocytes (Figure 6B). Similar with pattern in heart, the activity of FAK and AKT also decreased in Kcnh2^+/‐^ cardiomyocytes compared with WT rats challenged by LPS at different times (Figure 6C). To clarify whether AKT mediate the modulation of Kcnh2 during SICD, we treated Kcnh2^+/‐^ rats with bpV(HOpic), an activator of AKT, which has been proved to markedly enhance the phosphorylation level of AKT (Figure 6D). As we expected, the enhanced activity of AKT by bpV(HOpic) could significantly reduce heart tissue damage (Figure S10), and cardiac apoptosis combined with increase of FOXO3A and pro‐apoptotic genes BIM/PUMA as well as decrease of BCL‐2 in the Kcnh2^+/‐^ heart when challenged with LPS(Figure 6E‐F), that may be why AKT, at least, is one of potential regulators in FAK pathway to mediate the effect of Kcnh2 in protecting heart from sepsis damage.

## 4 DISCUSSION

Myocardial dysfunction occurs in the early stages of sepsis, which is the main cause of death in the intensive care unit.^3^ Our data suggest that Kcnh2 observed reductions in heart resulted from altered processing of cardiomyocytes, which, in turn, led to downregulation of the FAK/AKT activity, increasing the expression of FOXO3A and ultimately increasing pro‐apoptosis genes BIM/PUMA expression during sepsis. In addition, Kcnh2 activator NS1643 could improve cardiac function, suppressed the cardiomyocyte apoptosis. These results indicate that Kcnh2 will result in a protective effect on cardiac dysfunction during sepsis/septic shock.

Sepsis leads to high mortality associated with multiple organ injury, in which cardiac dysfunction is a well‐recognized manifestation that may attribute to metabolic abnormalities, mitochondrial dysfunction, inflammation, necrosis and apoptosis. Based on LPS or CLP surgery models, two general strategies to understand sepsis‐related cardiac dysfunction, we have previously found affected heart displayed a reduction of EF (%) and FS (%), accompanied with enhanced inflammatory response and increase of apoptosis in myocardium.^26^ SICD is involved in complex processes that associate with a variety of effectors. For example, eNOS and PLC**_λ_**1 signalling enhanced cardiac inflammatory response through stimulating TNF‐α expression.^27^, ^28^ Calpastatin could attenuate myocardial dysfunction by increasing caspase‐3 activation and TNF‐α expression during endotoxaemia.^29^ Incremental apelin‐13 bloodstream levels clearly improved myocardial performance through reducing release of inflammatory cytokine and apoptosis.^30^ Interestingly, recent studies revealed that potassium channels such as TWIK2 also involved in modulating LPS‐induced inflammatory injury during sepsis.^31^, ^32^ And growing number of work hinted that Kcnh2 also plays an important role in regulating of sepsis.^13^, ^14^, ^15^

Kcnh2 that encodes the voltage‐gated potassium channel was proved to exert a crucial role in cell fate determination.^33^ We and other studies have demonstrated that homozygous deletion of Kcnh2 can lead to cardiac developmental defects that finally result in embryonic lethality.^11^, ^34^ Inhibition of Kcnh2 by si‐RNA or inhibitor E4031 could remarkably attenuate cellular proliferation and migration due to inhibition the pathway of MAP kinase/c‐fos.^35^ Activation of Kcnh2 using activator NS1643, however, improved survival rate of breast cancer through a mechanism whereby inhibiting cell motility, attenuating Wnt/β‐catenin signalling to reprogramme epithelial–mesenchymal transition and suppressing cancer cell stemness.^36^ As a potential cytokine effector, Kcnh2 has been proved to be associated with sepsis related inflammatory processes.^15^, ^37^, ^38^, ^39^ We here found that Kcnh2 could be downregulated by LPS injection or CLP surgery. And its defect (Kcnh2^+/‐^) aggravated LPS‐induced cardiac dysfunction and decreased survival rate through inducing more apoptosis of cardiomyocytes. Activation of Kcnh2 by NS1643 exhibited opposite outcome, indicating a potential role of the molecule in heart protection during sepsis. However, the inflammatory response nearly has not significantly influenced by Kcnh2, which suggested its vital function on modulation of tolerance to sepsis in cardiomyocytes.

A growing body of evidences suggests that the PI3K/AKT pathway is involved in inflammatory response and apoptosis^40^ and plays an important role as a negative feedback regulator in excessive innate immune and Toll‐like receptor‐mediated pro‐inflammatory response.^17^, ^18^ Like previous observation, we found that LPS really induces activity of AKT by modulating its phosphorylation level in rat heart. Importantly, AKT could be also activated by Kcnh2 through interacting with integrin β1/FAK^41^, ^42^ or enhancing the phosphorylation of AKT independently.^20^ Integrin β1/FAK contributes to promote vascular leakage in endotoxaemia.^43^ When here induced a defect of Kcnh2 by genetic manipulation, the activity of both FAK and AKT was obviously impaired, which further aggravated the apoptosis due to LPS challenge. However, the situation was improved as long as activator bpV(HOpic) of AKT was applied, suggesting that Kcnh2 might mediate FAK/AKT signalling axis to modulate cardiomyocyte apoptosis during SICD.

FOXO3A, a member of the forkhead transcription factor family, is a major effector of AKT,^16^ and its activation can aggregate organ inflammatory damage and dysfunction via inducing the pro‐apoptosis gene BIM expression.^44^, ^45^ In our previous work, FOXO3A inhibition was demonstrated to significantly attenuate LPS‐induced cardiac damage,^26^, ^46^ so the inhibition of AKT activity due to defect of Kcnh2 (Kcnh2^+/‐^) may lead to upregulation of FOXO3A in the presence of LPS. As expected, we found LPS challenge induced an increase of FOXO3A in the myocardium of Kcnh2^+/‐^ rats, which correlated with the status of cardiac apoptosis; furthermore, the expression of pro‐apoptosis genes BIM and PUMA that were transcriptionally regulated by FOXO3A was also upregulated in LPS‐treated Kcnh2^+/‐^ rats. Moreover, inhibition of FOXO3A in Kcnh2‐defected cardiomyocytes could attenuate apoptosis and suppress BIM and PUMA expression. Thence, Kcnh2 can modulate AKT activity to affect the expression of FOXO3A in SICD.

## 5 CONCLUSIONS

**Figure 7:**
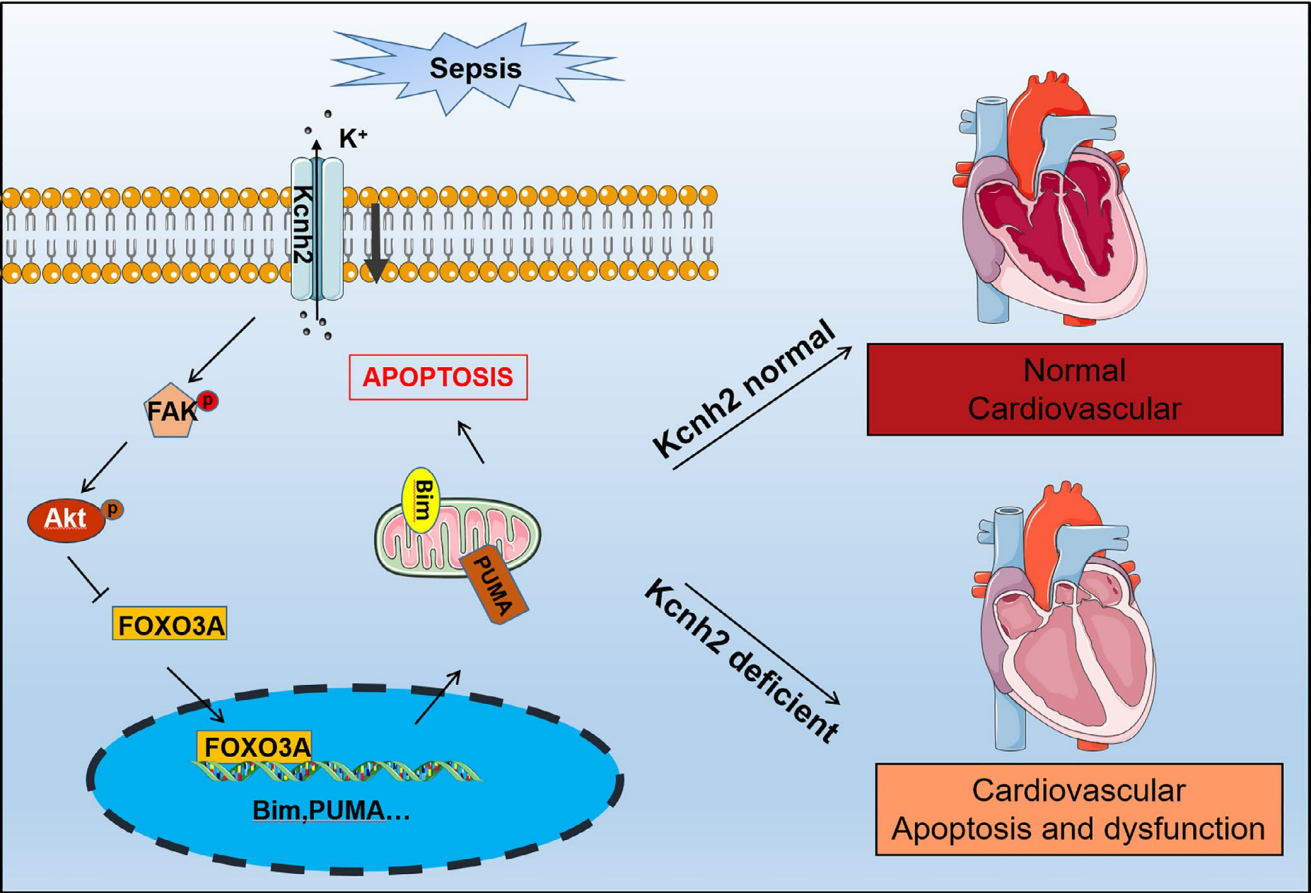
Schematic representation shows the mechanisms of Kcnh2‐modulated cardiac dysfunction following sepsis stimulus. Sepsis‐induced decrease of Kcnh2 resulted in inhibition of AKT in cardiomyocytes. Attenuation of AKT of cardiomyocytes mediated by Kcnh2 upregulated FOXO3A expression, which initiated the transcription of BIM/PUMA genes. Finally, enhanced BIM/PUMA caused the cardiomyocyte apoptosis, leading to cardiac dysfunction

Kcnh2 could negatively contribute to the SICD, at least in part, via FAK/AKT/FOXO3A pathway. Deficiency of Kcnh2 suppresses the phosphorylation of FAK and AKT, which, in turn, induces upregulation of their downstream target FOXO3A, resulting in an increase of BIM/PUMA expression. These results suggest a potential mechanism of Kcnh2 by which to involve apoptosis of cardiomyocytes and septic cardiac dysfunction (Figure 7).

## CONFLICT OF INTEREST

None declared.

## AUTHOR CONTRIBUTIONS

Luying Peng and Li Li conceived and supervised the project; Zhigang Li designed and performed the experiments. Zhigang Li and Chang Liu analysed the data; Yilei Meng, Huan Liu and Wenze Cao carried out part of experiments; Chang Tong and Min Lu were involved in the discussion of results. Zhigang Li wrote the manuscript. Luying Peng and Li Li revised the manuscript. All authors read and approved the manuscript.

## Supporting information

Figure S1

Figure S2

Figure S3

Figure S4

Figure S5

Figure S6

Figure S7

Figure S8

Figure S9

Figure S10

Figure S11

Supplementary information

## Data Availability

The data that support the findings of this study are available from the corresponding author upon reasonable request.

## References

[1] Hotchkiss RS , Nicholson DW . Apoptosis and caspases regulate death and inflammation in sepsis. Nat Rev Immunol. 2006;6(11):813‐822. 17039247 10.1038/nri1943

[2] Cohen J . The immunopathogenesis of sepsis. Nature. 2002;420:885‐891. 12490963 10.1038/nature01326

[3] Rudd KE , Johnson SC , Agesa KM , et al. Global, regional, and national sepsis incidence and mortality, 1990–2017: analysis for the Global Burden of Disease Study. Lancet. 2020;395(10219):200‐211. 31954465 10.1016/S0140-6736(19)32989-7 PMC6970225

[4] Sun Y , Yao X , Zhang QJ , et al. Beclin‐1‐Dependent autophagy protects the heart during sepsis. Circulation. 2018;138(20):2247‐2262. 29853517 10.1161/CIRCULATIONAHA.117.032821 PMC6274625

[5] An R , Zhao L , Xi C , et al. Melatonin attenuates sepsis‐induced cardiac dysfunction via a PI3K/Akt‐dependent mechanism. Basic Res Cardiol. 2016;111(1):8. 26671026 10.1007/s00395-015-0526-1

[6] Knuefermann P , Nemoto S , Misra A , et al. CD14‐deficient mice are protected against lipopolysaccharide‐induced cardiac inflammation and left ventricular dysfunction. Circulation. 2002;106(20):2608‐2615. 12427659 10.1161/01.cir.0000038110.69369.4c

[7] Nezic L , Skrbic R , Amidzic L , Gajanin R , Kuca K , Jacevic V . Simvastatin protects cardiomyocytes against endotoxin‐induced apoptosis and up‐regulates survivin/NF‐kappaB/p65 expression. Sci Rep. 2018;8(1):e14652. 10.1038/s41598-018-32376-4 PMC6168467 30279549

[8] Lorinczi E , Gomez‐Posada JC , de la Pena P , et al. Voltage‐dependent gating of KCNH potassium channels lacking a covalent link between voltage‐sensing and pore domains. Nat Commun. 2015;6:6672. 25818916 10.1038/ncomms7672 PMC4389246

[9] Anderson CL , Delisle BP , Anson BD , et al. Most LQT2 mutations reduce Kv11.1 (hERG) current by a class 2 (trafficking‐deficient) mechanism. Circulation. 2006;113(3):365‐373. 16432067 10.1161/CIRCULATIONAHA.105.570200

[10] Jehle J , Schweizer PA , Katus HA , Thomas D . Novel roles for hERG K(+) channels in cell proliferation and apoptosis. Cell Death Dis. 2011;2:e193. 21850047 10.1038/cddis.2011.77 PMC3181422

[11] Teng GQ , Zhao X , Lees‐Miller JP , et al. Homozygous missense N629D hERG (KCNH2) potassium channel mutation causes developmental defects in the right ventricle and its outflow tract and embryonic lethality. Circ Res. 2008;103(12):1483‐1491. 18948620 10.1161/CIRCRESAHA.108.177055 PMC2774899

[12] Staudacher I , Jehle J , Staudacher K , et al. HERG K+ channel‐dependent apoptosis and cell cycle arrest in human glioblastoma cells. PLoS One. 2014;9(2):e88164. 24516604 10.1371/journal.pone.0088164 PMC3916397

[13] Aoki Y , Hatakeyama N , Yamamoto S , et al. Role of ion channels in sepsis‐induced atrial tachyarrhythmias in guinea pigs. Br J Pharmacol. 2012;166(1):390‐400. 22050008 10.1111/j.1476-5381.2011.01769.x PMC3415663

[14] Zila IMD , Kopincova J , Kolomaznik M , Javorka M , Calkovska A . Heart rate variability and inflammatory response in rats with lipopolysaccharide‐induced endotoxemia. Physiol Res. 2015;64:S669‐S676. 26674290 10.33549/physiolres.933226

[15] Aromolaran AS , Srivastava U , Ali A , et al. Interleukin‐6 inhibition of hERG underlies risk for acquired long QT in cardiac and systemic inflammation. PLoS One. 2018;13(12):e0208321. 30521586 10.1371/journal.pone.0208321 PMC6283635

[16] Anne Brunet AB , Zigmond MJ , Lin MZ , et al. Akt promotes cell survival by phosphorylating and inhibiting a Forkhead transcription factor. Cell. 1999;96:857‐868. 10102273 10.1016/s0092-8674(00)80595-4

[17] Fattahi F , Kalbitz M , Malan EA , et al. Complement‐induced activation of MAPKs and Akt during sepsis: role in cardiac dysfunction. FASEB J. 2017;31(9):4129‐4139. 28572445 10.1096/fj.201700140R PMC5572692

[18] Schabbauer G , Tencati M , Pedersen B , Pawlinski R , Mackman N . PI3K‐Akt pathway suppresses coagulation and inflammation in endotoxemic mice. Arterioscler Thromb Vasc Biol. 2004;24(10):1963‐1969. 15319270 10.1161/01.ATV.0000143096.15099.ce

[19] Zhang X , Li N , Shao H , et al. Methane limit LPS‐induced NF‐kappaB/MAPKs signal in macrophages and suppress immune response in mice by enhancing PI3K/AKT/GSK‐3beta‐mediated IL‐10 expression. Sci Rep. 2016;6:e29359. 10.1038/srep29359 PMC4942692 27405597

[20] Becchetti A , Petroni G , Arcangeli A . Ion channel conformations regulate integrin‐dependent signaling. Trends Cell Biol. 2019;29(4):298‐307. 30635161 10.1016/j.tcb.2018.12.005

[21] Skurk C , Maatz H , Kim HS , et al. The Akt‐regulated forkhead transcription factor FOXO3a controls endothelial cell viability through modulation of the caspase‐8 inhibitor FLIP. J Biol Chem. 2004;279(2):1513‐1525. 14551207 10.1074/jbc.M304736200

[22] Schips TG , Wietelmann A , Hohn K , et al. FoxO3 induces reversible cardiac atrophy and autophagy in a transgenic mouse model. Cardiovasc Res. 2011;91(4):587‐597. 21628326 10.1093/cvr/cvr144

[23] Chaanine AH , Jeong D , Liang L , et al. JNK modulates FOXO3a for the expression of the mitochondrial death and mitophagy marker BNIP3 in pathological hypertrophy and in heart failure. Cell Death Dis. 2012;3:265. 22297293 10.1038/cddis.2012.5 PMC3288347

[24] Rittirsch D , Huber‐Lang MS , Flierl MA , Ward PA . Immunodesign of experimental sepsis by cecal ligation and puncture. Nat Protoc. 2009;4(1):31‐36. 19131954 10.1038/nprot.2008.214 PMC2754226

[25] Luan H , Wang A , Hilliard B , et al. GDF15 is an inflammation‐induced central mediator of tissue tolerance. Cell. 2019;178(5):1231‐1244.e11. 31402172 10.1016/j.cell.2019.07.033 PMC6863354

[26] Li Z , Zhu H , Liu C , et al. GSK‐3beta inhibition protects the rat heart from the lipopolysaccharide‐induced inflammation injury via suppressing FOXO3A activity. J Cell Mol Med. 2019;23(11):7796‐7809. 31503410 10.1111/jcmm.14656 PMC6815822

[27] Peng T , Lu X , Lei M , Feng Q . Endothelial nitric‐oxide synthase enhances lipopolysaccharide‐stimulated tumor necrosis factor‐alpha expression via cAMP‐mediated p38 MAPK pathway in cardiomyocytes. J Biol Chem. 2003;278(10):8099‐8105. 12506117 10.1074/jbc.M207288200

[28] Peng T , Shen E , Fan J , Zhang Y , Arnold JM , Feng Q . Disruption of phospholipase Cgamma1 signalling attenuates cardiac tumor necrosis factor‐alpha expression and improves myocardial function during endotoxemia. Cardiovasc Res. 2008;78(1):90‐97. 18079103 10.1093/cvr/cvm100

[29] Li X , Li Y , Shan L , Shen E , Chen R , Peng T . Over‐expression of calpastatin inhibits calpain activation and attenuates myocardial dysfunction during endotoxaemia. Cardiovasc Res. 2009;83(1):72‐79. 19318376 10.1093/cvr/cvp100

[30] Chagnon F , Coquerel D , Salvail D , et al. Apelin compared with dobutamine exerts cardioprotection and extends survival in a rat model of endotoxin‐induced myocardial dysfunction. Crit Care Med. 2017;45(4):e391‐e398. 27571457 10.1097/CCM.0000000000002097

[31] Di A , Xiong S , Ye Z , et al. The TWIK2 potassium efflux channel in macrophages mediates NLRP3 inflammasome‐induced inflammation. Immunity. 2018;49(1):56‐65.e54. 29958799 10.1016/j.immuni.2018.04.032 PMC6051907

[32] Munoz‐Planillo R , Kuffa P , Martinez‐Colon G , Smith BL , Rajendiran TM , Nunez G . K(+) efflux is the common trigger of NLRP3 inflammasome activation by bacterial toxins and particulate matter. Immunity. 2013;38(6):1142‐1153. 23809161 10.1016/j.immuni.2013.05.016 PMC3730833

[33] Babcock JJ , Li M . hERG channel function: beyond long QT. Acta Pharmacol Sin. 2013;34(3):329‐335. 23459091 10.1038/aps.2013.6 PMC3587915

[34] Teng G , Zhao X , Lees‐Miller JP , et al. Role of mutation and pharmacologic block of human KCNH2 in vasculogenesis and fetal mortality partial rescue by transforming growth factor‐β. Circ Arrhythm Electrophysiol. 2015;8(2):420‐428. 25648353 10.1161/CIRCEP.114.001837

[35] Afrasiabi E , Hietamaki M , Viitanen T , Sukumaran P , Bergelin N , Tornquist K . Expression and significance of HERG (KCNH2) potassium channels in the regulation of MDA‐MB‐435S melanoma cell proliferation and migration. Cell Signal. 2010;22(1):57‐64. 19765650 10.1016/j.cellsig.2009.09.010

[36] Breuer EK , Fukushiro‐Lopes D , Dalheim A , et al. Potassium channel activity controls breast cancer metastasis by affecting beta‐catenin signaling. Cell Death Dis. 2019;10(3):180. 30792401 10.1038/s41419-019-1429-0 PMC6385342

[37] Kawada H , Niwano S , Niwano H , et al. Tumor necrosis factor‐α downregulates the voltage gated outward K+ current in cultured neonatal rat cardiomyocytes. Circulation J. 2006;70(5):605‐609. 10.1253/circj.70.605 16636498

[38] Wang J , Wang H , Zhang Y , Gao H , Nattel S , Wang Z . Impairment of HERG K(+) channel function by tumor necrosis factor‐alpha: role of reactive oxygen species as a mediator. J Biol Chem. 2004;279(14):13289‐13292. 14973143 10.1074/jbc.C400025200

[39] Monnerat G , Alarcon ML , Vasconcellos LR , et al. Macrophage‐dependent IL‐1beta production induces cardiac arrhythmias in diabetic mice. Nat Commun. 2016;7:e13344. 10.1038/ncomms13344 PMC5123037 27882934

[40] Manning BD , Cantley LC . AKT/PKB signaling: navigating downstream. Cell. 2007;129(7):1261‐1274. 17604717 10.1016/j.cell.2007.06.009 PMC2756685

[41] Cherubini A . Human ether‐a‐go‐go‐related gene 1 channels are physically linked to β1 integrins and modulate adhesion‐dependent signaling. Mol Biol Cell. 2005;16:2972‐2983. 15800067 10.1091/mbc.E04-10-0940 PMC1142440

[42] Arcangeli A , Becchetti A . Complex functional interaction between integrin receptors and ion channels. Trends Cell Biol. 2006;16(12):631‐639. 17064899 10.1016/j.tcb.2006.10.003

[43] Hakanpaa L , Kiss EA , Jacquemet G , et al. Targeting beta1‐integrin inhibits vascular leakage in endotoxemia. Proc Natl Acad Sci USA. 2018;115(28):E6467‐E6476. 29941602 10.1073/pnas.1722317115 PMC6048499

[44] Stahl M , Dijkers PF , Kops GJPL , et al. The Forkhead transcription factor FoxO regulates transcription of p27Kip1 and Bim in response to IL‐2. J Immunol. 2002;168(10):5024‐5031. 11994454 10.4049/jimmunol.168.10.5024

[45] Zhu S , Evans S , Yan B , et al. Transcriptional regulation of Bim by FOXO3a and Akt mediates scleroderma serum‐induced apoptosis in endothelial progenitor cells. Circulation. 2008;118(21):2156‐2165. 18981303 10.1161/CIRCULATIONAHA.108.787200 PMC3719010

[46] Li Z , Yi N , Chen R , et al. miR‐29b‐3p protects cardiomyocytes against endotoxin‐induced apoptosis and inflammatory response through targeting FOXO3A. Cell Signal. 2020;74:e109716. 10.1016/j.cellsig.2020.109716 32707074

