# Supplementary figures and images for: Kcnh2 mediates FAK/AKT‐FOXO3A pathway to attenuate sepsis‐induced cardiac dysfunction
Source: Cell Prolif. 2020 Dec 2;54(2):e12962. doi: 10.1111/cpr.12962 (PMC7848965; doi:10.1111/cpr.12962)

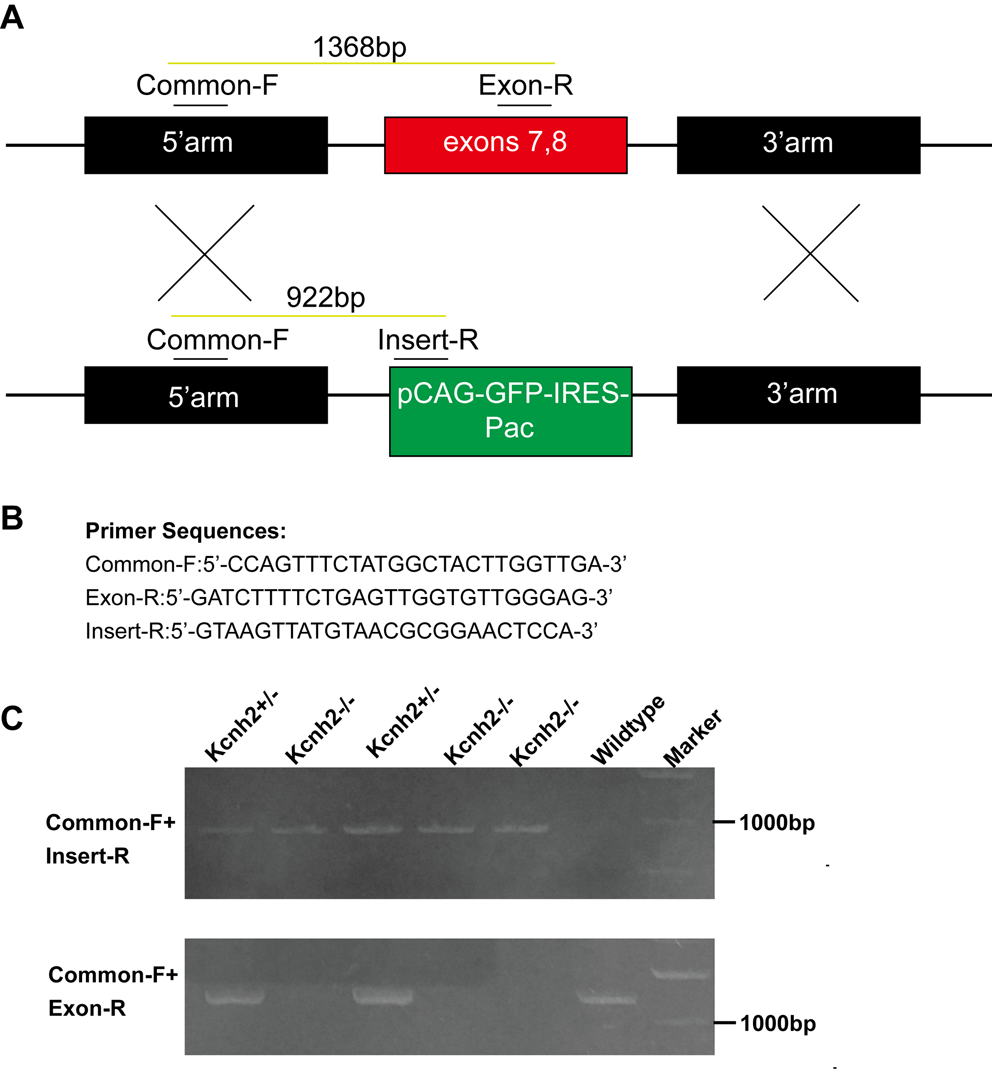

Supplement: Supplementary file 1 — Figure S1 [file CPR-54-e12962-s001.tif]

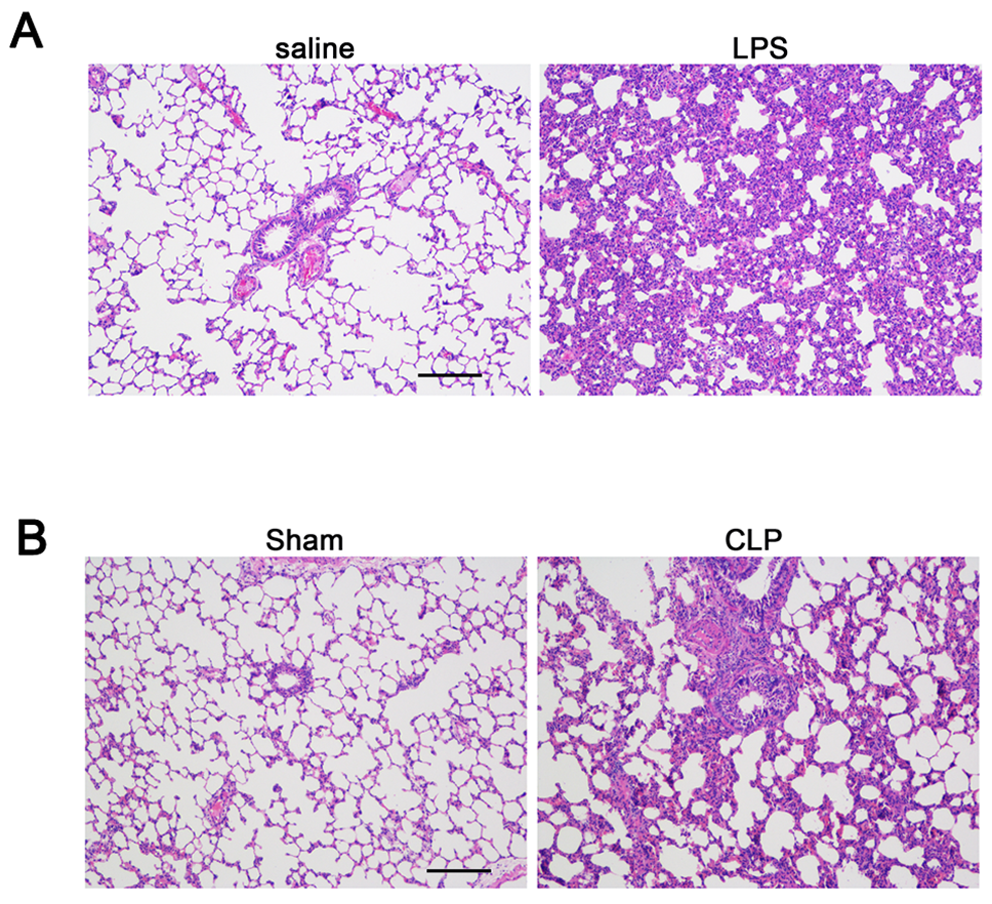

Supplement: Supplementary file 2 — Figure S2 [file CPR-54-e12962-s002.tif]

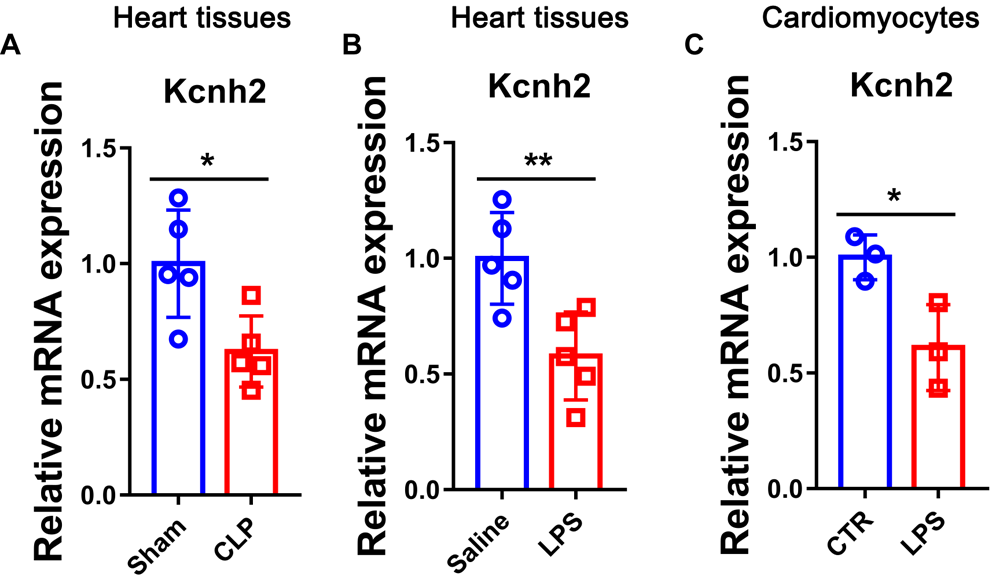

Supplement: Supplementary file 3 — Figure S3 [file CPR-54-e12962-s003.tif]

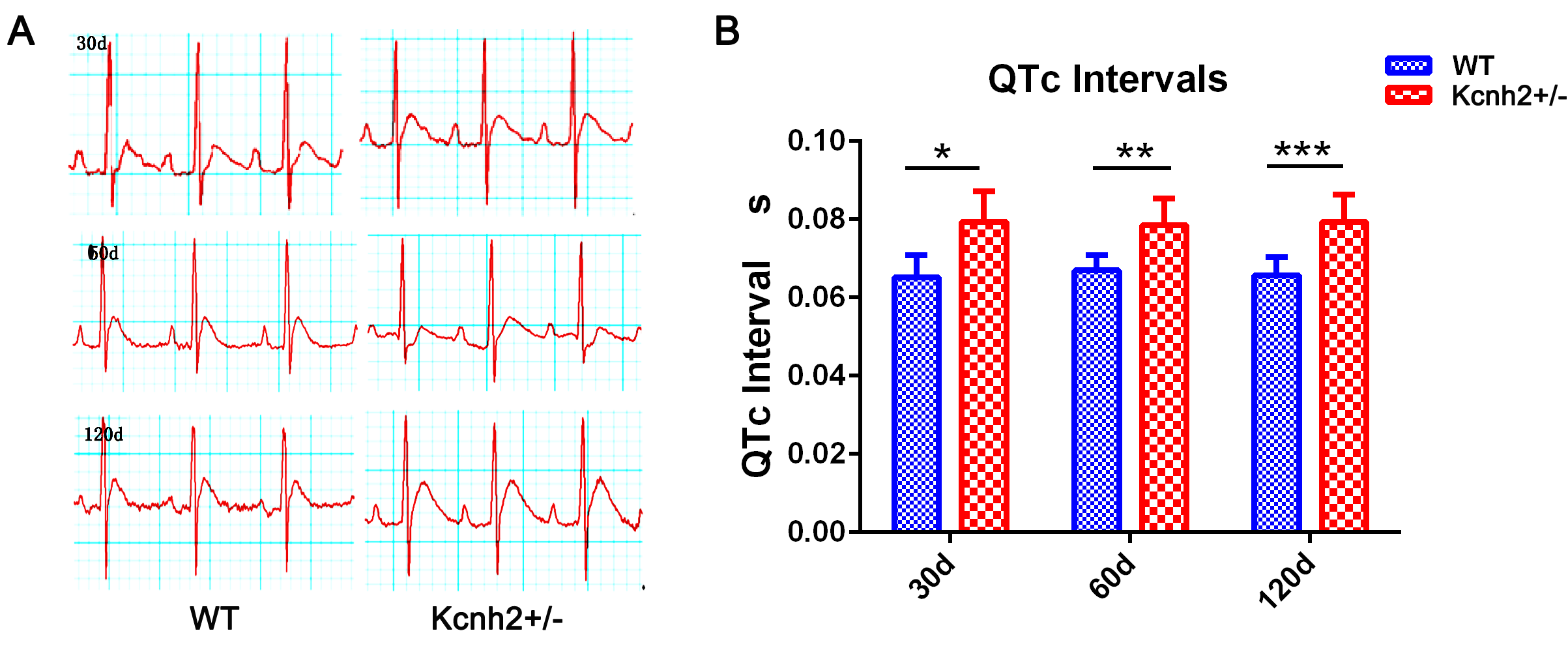

Supplement: Supplementary file 4 — Figure S4 [file CPR-54-e12962-s004.tif]

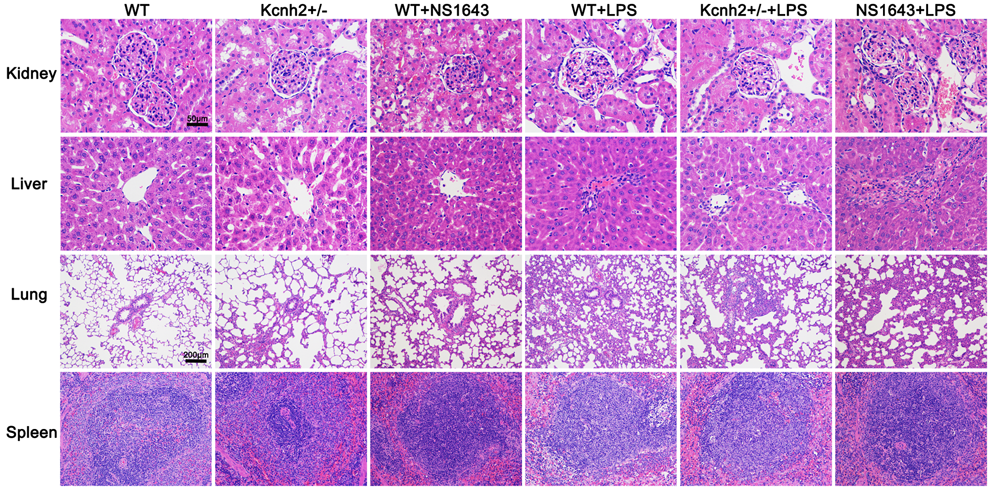

Supplement: Supplementary file 5 — Figure S5 [file CPR-54-e12962-s005.tif]

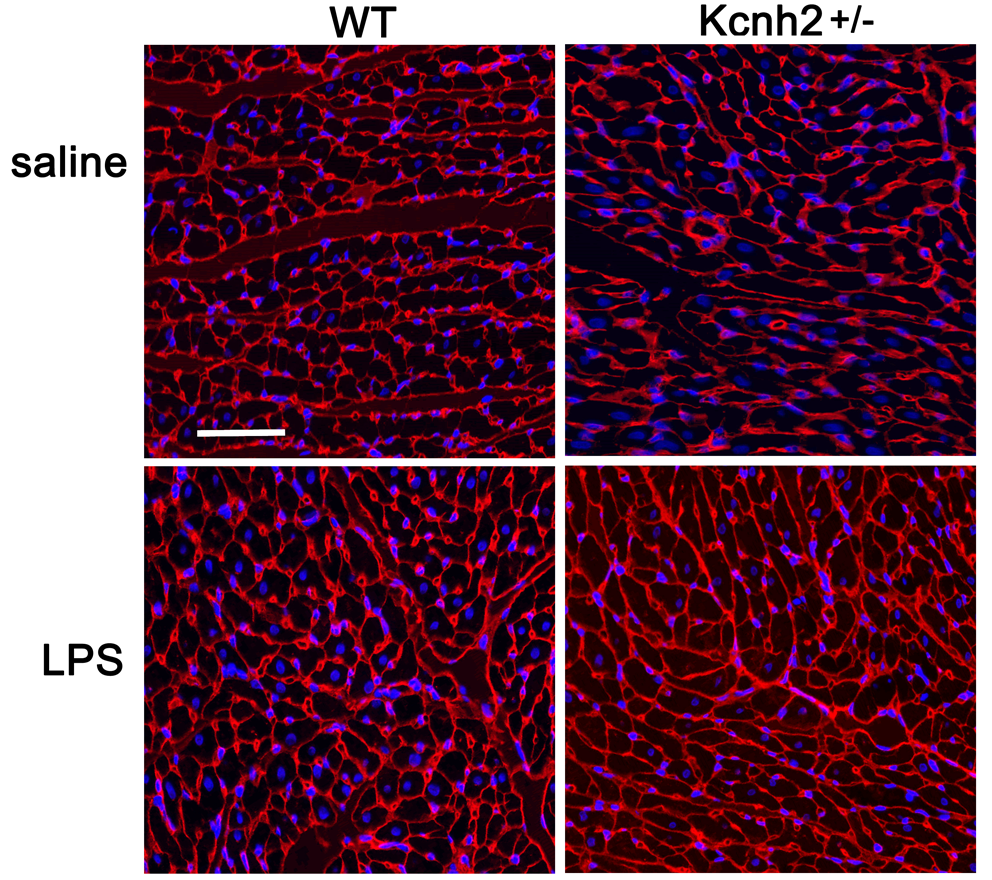

Supplement: Supplementary file 6 — Figure S6 [file CPR-54-e12962-s006.tif]

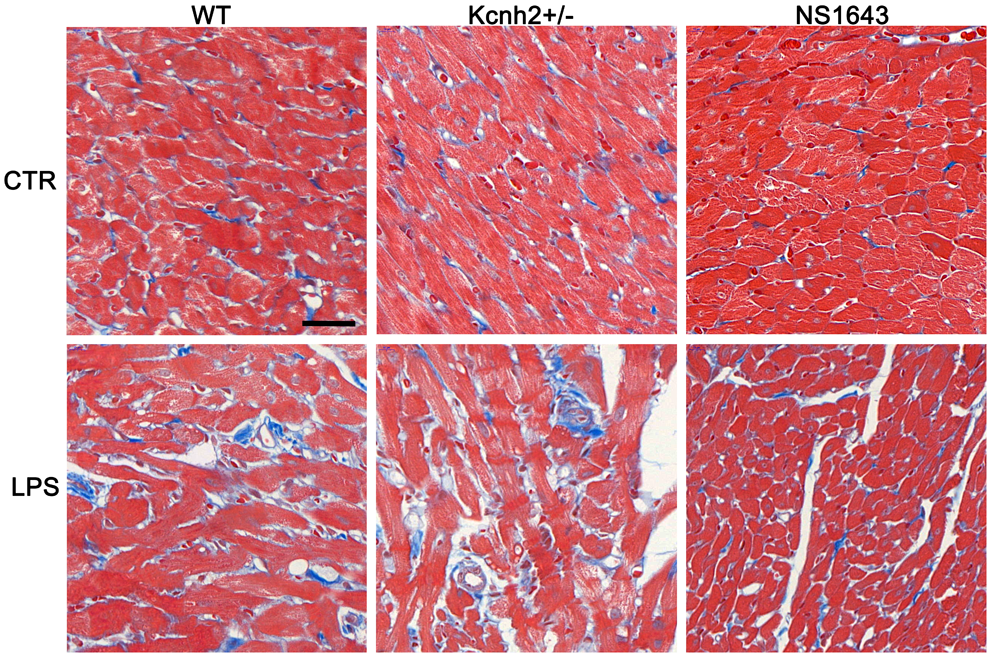

Supplement: Supplementary file 7 — Figure S7 [file CPR-54-e12962-s007.tif]

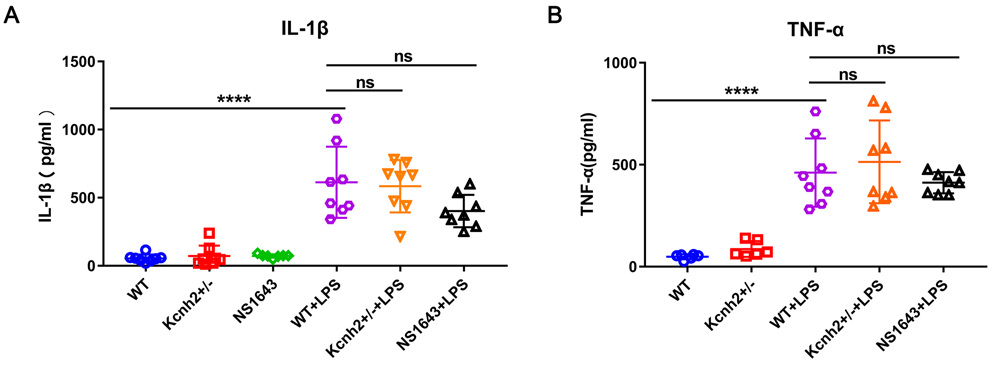

Supplement: Supplementary file 8 — Figure S8 [file CPR-54-e12962-s008.tif]

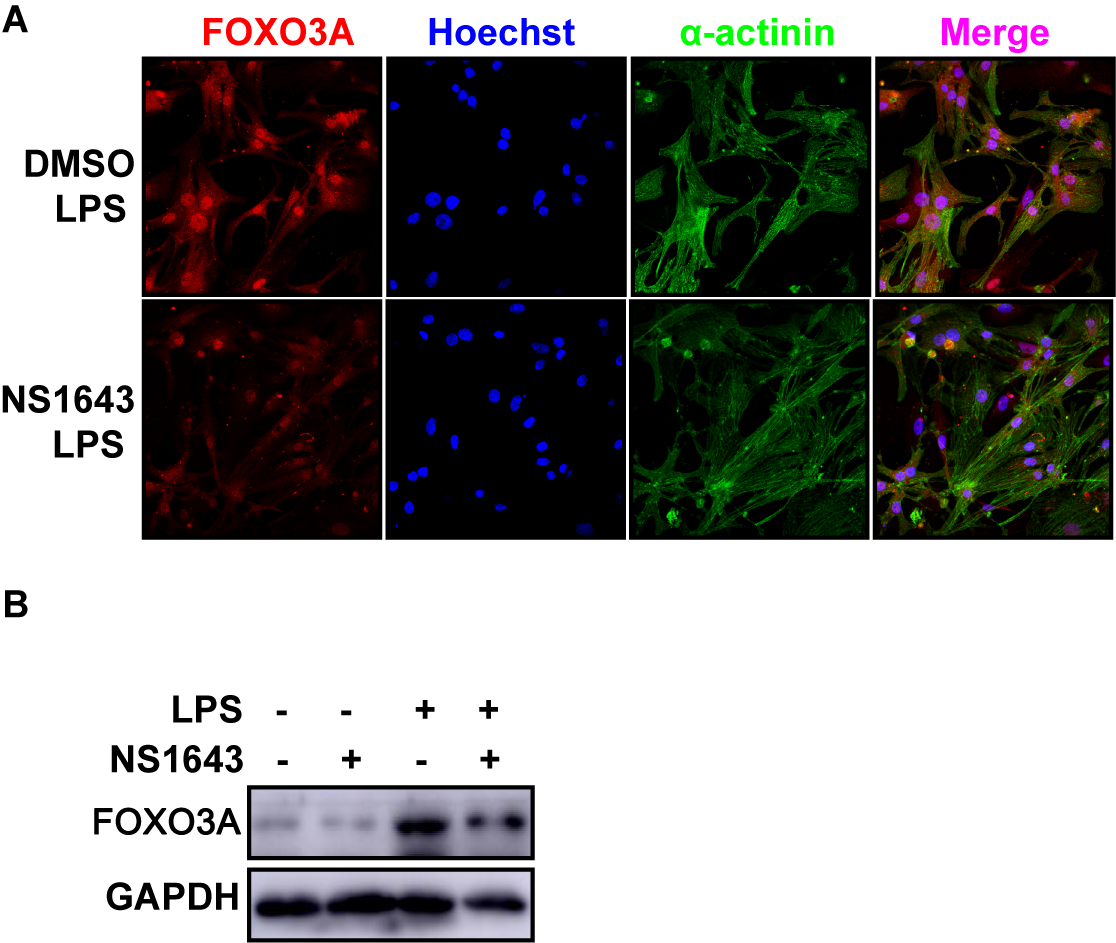

Supplement: Supplementary file 9 — Figure S9 [file CPR-54-e12962-s009.tif]

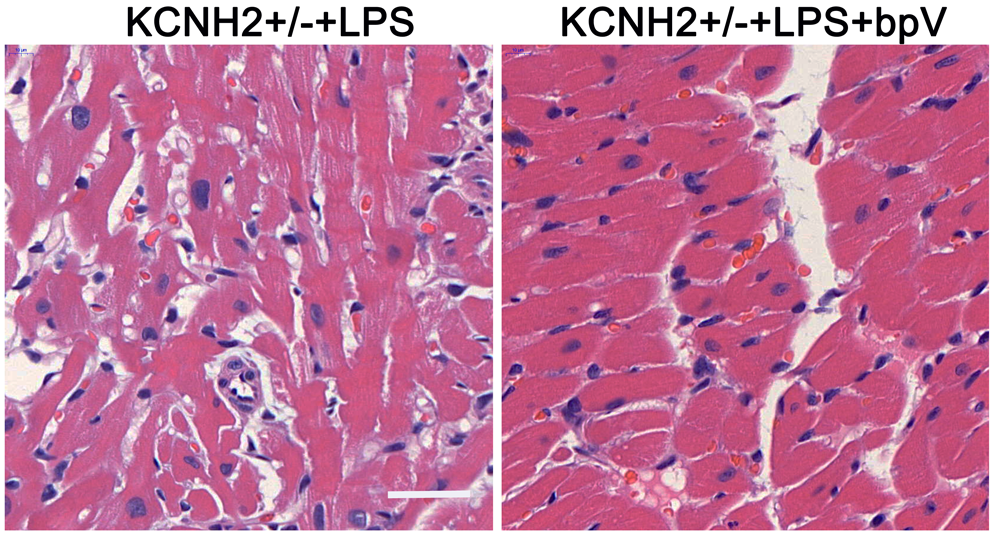

Supplement: Supplementary file 10 — Figure S10 [file CPR-54-e12962-s010.tif]

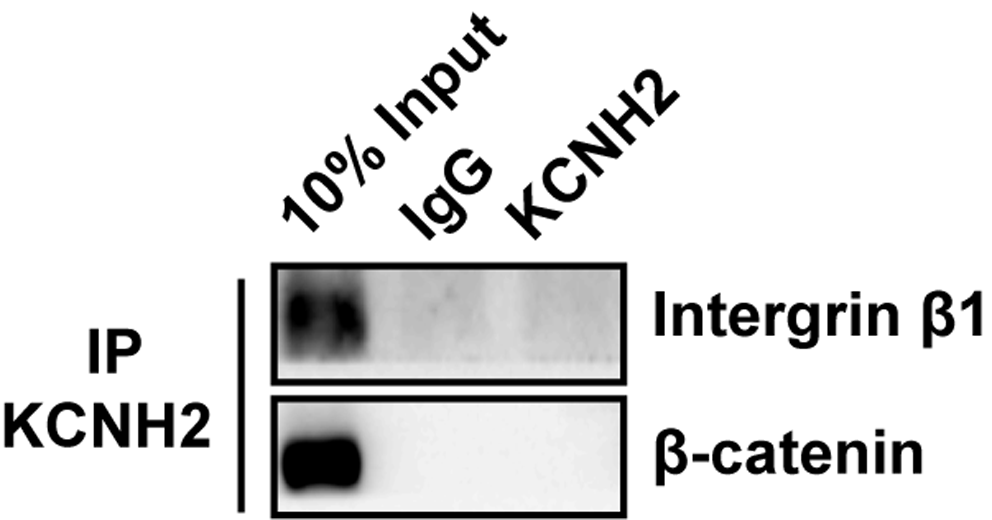

Supplement: Supplementary file 11 — Figure S11 [file CPR-54-e12962-s011.tif]
