# Supplementary material for: Kcnh2 mediates FAK/AKT‐FOXO3A pathway to attenuate sepsis‐induced cardiac dysfunction
Source: Cell Prolif. 2020 Dec 2;54(2):e12962. doi: 10.1111/cpr.12962 (PMC7848965; doi:10.1111/cpr.12962)
Supplement: Supplementary file 12 — Supplementary information [file CPR-54-e12962-s012.docx]

**Title: Kcnh2 mediates FAK/AKT-FOXO3A pathway to attenuate sepsis-induced cardiac dysfunction**

**Short title: Kcnh2 in Septic Hearts**

Zhigang Li^1,2,3,5^, Yilei Meng^1,2,3,5^, Chang Liu^1,2,3^, Huan Liu^1,2,3,5^, Wenze Cao^1,2,3,5^, Chang Tong^3^, Min Lu^3^, Li Li^1,2,3,4,5*^, Luying Peng^1,2,3,4,5*^

^1^Key Laboratory of Arrhythmias, Ministry of Education, Shanghai East Hospital, Tongji University School of Medicine, Shanghai 200120

^2^Institute of Medical Genetics, Tongji University, Shanghai 200092

^3^Heart Health Center, Shanghai East Hospital, Tongji University School of Medicine, Shanghai 200120 China

^4^Research Units of Origin and Regulation of Heart Rhythm, Chinese Academy of Medical Sciences, Beijing, 100730 China.

^5^Department of Medical Genetics, Tongji University School of Medicine, Shanghai 200092

*Correspondence: Luying Peng, E-mail: [luyingpeng@tongji.edu.cn](mailto:luyingpeng@tongji.edu.cn)，Tel: +86-21-65983617，Fax: 086-21-65983607， Key Laboratory of Arrhythmias, Ministry of Education, Shanghai East Hospital, Tongji University School of Medicine or

Li Li, E-mail: lilirz@tongji.edu.cn，Tel: +86-21-65983617，Fax: 086-21-65983607，Key Laboratory of Arrhythmias, Ministry of Education, Shanghai East Hospital, Tongji University School of Medicine

**Figure legends**

Figure S1 Development of Kcnh2 transgenic rats

(A) Construct of Kcnh2 transgene. (B) Gene typing primers. (C) Transmission of Kcnh2 transgene to offspring was genotyped by PCR.

Figure S2 Sepsis caused lung injury

Hematoxylin–eosin staining (H&E staining) was performed to detect lung injury, a typical phenotype of the sepsis model induced by CLP surgery and LPS treated. Scale bar: 100μm. At 6 h after induction, (n=6).

Figure S3 Sepsis reduces myocardial Kcnh2 expression

Qpcr was used to analyize the expression of Kcnh2 in (A) CLP surgery and (B)LPS injection rat heart; (C)LPS treated cardiomyocyte.

Figure S4 Kcnh2 knockout causes prolong of QTs internal

Electrocardiogram (ECG) was performed to detect the change of heart rhythm. After birth of 30d, 60d and 120d, the electrical activity of the Kcnh2+/- group and WT group were detected by 3-lead electrocardiogram (ECG) (n≥3).

Figure S5 Effect of Kcnh2 on damage of Kidney, Liver, Lung and Spleen induced by LPS.

H&E staining was performed to detect damage of Kidney, Liver (Scale bar: 50μm), Lung and Spleen (Scale bar: 200μm) at 6 h after LPS induction of wildtype, Kcnh2^+/-^ and NS1643 (n=6).

Figure S6 Kcnh2 knockout aggregates LPS-induced cardiomyocyte edema

WAG staining was used to analyze the change of hypertrophy in Kcnh2+/- rat by LPS challenge.

Figure S7 Regulation of Kcnh2 on LPS-stimulated fibrosis of rat heart

Histological trichrome (Masson) stain was applied to the heart tissue sections to visualize collagenous connective tissue fibers (blue). At 6 h after LPS induction of wildtype, Kcnh2^+/-^ and NS1643 (n=6).

Figure S8 Effect of Kcnh2 on the production of IL-1β and TNF-α induced by LPS

Cytokines, IL-1β and TNF-α were measured in the heart tissue lysates by multiplex ELISA assays. At 6 h after LPS induction of wildtype, Kcnh2^+/-^ and NS1643 (n=6).

Figure S9 NS1643 modulates the expression of FOXO3A expression in sepsis

(A)Immunofluorescence analysis of the influence of NS1643 on FOXO3A expression after LPS treatment.(B)Western blotting detected the expression of FOXO3A.

Figure S10 bpV(HOpic) attenuates the cardiac damage induced by LPS

H&E staining for detecting the heart tissue damage induced by LPS in NRCMs pre-treated with bpV(HOpic) at 6mg/kg in Kcnh2^+/-^ rats. (n=5).

Figure S11 KCNH2 have no interaction with intergrin β1/β-cateiin

Co-IP assay was performed to detected interaction between KCNH2 and intergrin β1/β-catenin in rat cardiomyocyte.
